# Stopping or maintaining oral anticoagulation in patients undergoing photoselective vaporization of the prostate (SOAP) surgery for benign prostate obstruction: study protocol for a multicentre randomized controlled trial

**DOI:** 10.1186/s13063-018-3066-9

**Published:** 2018-12-27

**Authors:** Hélène Charbonneau, Marie Pasquié, Benoit Peyronnet, Aurélien Descazeaud, Nicolas Barry-Delongchamps, Emmanuel Della Negra, Romain Mathieu, Gilles Karsenty, Jean-Alexandre Long, Charles Ballereau, Abdel-Rahmène Azzouzi, Benjamin Pradère, Franck Bruyère, Georges Fournier, Souhil Lebdai, Jehanne Calves, Luc Corbel, Sébastien Vincendeau, Gaelle Fiard, Caroline Thuillier, Jean-Luc Descotes, Pierre Colin, Thibaut Culty, Audrey Hesbois, Valerie Fuzier, Nicolas Savy, Atul Pathak, Pierre Albaladejo, Charles Marc Samama, Felipe Guerrero, Vincent Misraï

**Affiliations:** 1 Department of Anesthesia, Clinique Pasteur, Toulouse, France; 2 Department of Urology, Clinique Pasteur, 45 avenue de Lombez, BP 27 617, 31076 Toulouse Cedex 3, France; 3 Department of Urology, Rennes University Hospital , Rennes, France; 4 Department of Urology, Limoges University Hospital, Dupuytren Hospital, Limoges, France; 5 Department of Urology, Cochin University Hospital, Paris, France; 6 Department of Urology, Clinique Privée des Cotes d’Armor, Plerin, France; 7 Department of Urology, Conception Hospital, Marseille, France; 8 Department of Urology, Grenoble University Hospital , Michallon Hospital, La Tronche, France; 9 Department of Urology, Hopital Privée la Louviere, Lille, France; 10 Department of Urology, Angers University Hospital , Angers, France; 11 Department of Urology, Tours University Hospital , Tours, France; 12 Department of Urology, Brest University Hospital, Cavale Blanche Hospital, Brest, France; 13 Institute of mathematics, Paul Sabatier University, CNRS, Toulouse, France; 14 Department of Cardiovascular Medicine, Clinique Pasteur, Toulouse, France; 15 Department of Anaesthesia and Intensive Care, Grenoble University Hospital, Avenue Maquis-du-Grésivaudan, 38700 La Tronche, France; 16 Clinical Investigation Centre, Grenoble University Hospital, ThEMAS, TIMC, UMR-CNRS 5525, University Grenoble-Alpes, 38700 La Tronche, France; 17 Department of Anaesthesiology and Intensive Care, Assistance publique-Hôpitaux de Paris, Cochin University Hospital, 75014 Paris, France; 18 Department of Haematology, Toulouse University Hospital , Rangueil, France

**Keywords:** Benign prostatic obstruction, GreenLight, Laser, Oral anticoagulation, Complications

## Abstract

**Background:**

Lower urinary tract symptoms related to benign prostatic obstruction (BPO) are frequent in men aged > 50 years. Based on the use of innovative medical devices, a number of transurethral ablative techniques have recently been developed for the surgical treatment of BPO. In recent years, GreenLight photoselective vaporization of the prostate (PVP) has been considered as a non-inferior alternative to transurethral resection of the prostate. The GreenLight PVP is usually considered as an interesting surgical option for patients treated via oral anticoagulants (OACs) with regard to its haemostatic properties. The aim of this study was to assess the impact of maintaining OAC treatment in patients undergoing PVP.

**Methods:**

This study is a multicentre, open-label, randomized controlled trial (RCT) designed to show the non-inferiority of PVP surgery in patients with BPO treated with OACs. This study is designed to enrol 386 OAC-treated patients (treated with vitamin K antagonists and direct oral anticoagulants) who are undergoing PVP for BPO. Patients will be randomized (1:1) to either maintain or stop OAC treatment during the perioperative course. The intervention group will maintain OAC treatment until the day before surgery and resume OAC treatment the day after surgery, whereas the control group will stop OAC treatment (with or without low-molecular-weight heparin bridging therapy) according to the anaesthesia guidelines. The primary outcome of interest to be assessed is the 30-day complications rate according to the Clavien-Dindo classification. The secondary endpoint will examine the 30-day rate of haemorrhagic and thrombotic events. This study will provide 80% power to show non-inferiority, defined as not worse than a 10% (non-inferiority margin) inferior change in the proportion of patients with good outcomes (Clavien-Dindo score < 2), using two-tailed 95% confidence intervals.

**Discussion:**

This first multicentre RCT in the field is underway to evaluate the safety and efficacy of PVP in patients with ongoing OAC therapy. The study results could influence the perioperative management of OACs in BPO surgery with a high level of evidence.

**Trial registration:**

ClinicalTrials.gov, NCT03297281. Registered on 29 September 2017.

## Background

Urologists and anaesthesiologists are routinely challenged by the surgical management of men with benign prostatic obstruction (BPO) who are taking oral anticoagulants (OACs) [1]. The number of patients admitted for surgery who are treated with vitamin K antagonists (VKAs) and direct oral anticoagulants (DOACs) continues to rise. To improve the safety of ageing and frail patients treated with OACs, new “minimally invasive” surgical techniques have recently emerged [2].

Photoselective vaporization of the prostate (PVP) with the GreenLight 532-nm laser (Boston Scientific Corporation, Marlborough, MA, USA) is one of the fastest growing alternatives to transurethral resection of the prostate (TURP) for prostates < 80 ml in the last decade [3]. Initially, GreenLight PVP was introduced as an 80-W system, and increasingly high-powered lasers have since been developed. Several meta-analyses of the 80-W and 120-W high-performance systems (HPSs) report that PVP has similar efficacy and reduced complication rates compared to TURP [4, 5]. In addition, a recent randomized controlled trial (RCT), the “Goliath Study,” reported that PVP using the new 180-W generator Xcelerated Performance System (XPS) provided non-inferior functional outcomes compared to TURP with significantly reduced bleeding complication rates [6–8]. To date, an average of 30% of BPO surgeries are carried out in France using the 180-W XPS laser [9].

In regard to the haemostasis properties of the 532-nm laser [10], several studies have retrospectively investigated the feasibility of PVP without bridging or interrupting OAC treatment, but the amount of evidence remains low and relies on poorly designed studies [11, 12]. Moreover, the consequences of OAC perioperative management on PVP surgical morbidity have never been evaluated in a randomized study. Our objective was to assess the impact of maintaining OAC treatment on perioperative morbidity in patients undergoing PVP.

## Methods/design

### Study design

Stopping or maintaining oral anticoagulation in patients undergoing PVP (SOAP) is a randomized, open-label, multicentre, non-inferiority trial with two parallel groups. Patients will be allocated 1:1 to either the intervention group (S1) or the control group (S2). This study will be conducted in 11 centres in France. This study seeks to assess whether maintenance of OAC treatment increases the risk of adverse events (AEs) in patients undergoing PVP. Postoperative complications will be identified according to the Clavien-Dindo classification [13, 14]. This trial is registered with ClinicalTrials.gov (NCT03297281).

### Inclusion criteria

The criteria for inclusion are as follows: patients with a prostate volume ≤ 80 ml (documented via ultrasonography); patients with bothersome lower urinary tract symptoms (documented with an International Prostate Symptoms Score [IPSS] and uroflowmetry) that are refractory to medical treatment and/or those who have complications related to BPO; patients who are candidates for PVP; patients treated with a VKA for 3 months or longer and with an international normalized ratio (INR) therapeutic range from 2 to 3 or treated with a DOAC for 3 months or longer; patients who are able to comply with all study requirements and have signed a study-specific informed consent form.

### Exclusion criteria

The exclusion criteria for this study are history of prostate cancer, previous pelvic radiotherapy, or stenosis of the urethra; bladder tumour; treatment with antiplatelet agents (other than aspirin); allergy to heparin or history of heparin-induced thrombocytopenia; treatment with an injectable anticoagulant treatment at baseline (e.g. heparin, low-molecular-weight-heparin [LMWH], fondaparinux); mechanical prosthetic heart valve; stroke (ischaemic or haemorrhagic), systemic embolism or transient ischaemic attack (TIA) within past 12 weeks; venous thromboembolism (deep vein thrombosis and/or pulmonary embolism) within past 12 weeks; major bleeding within past 6 weeks; severe renal insufficiency (calculated creatinine clearance < 30 ml/min); thrombocytopenia (platelet count < 100 × 10^9^/L); life expectancy < 1 month; a condition that impairs compliance with the trial protocol (e.g. cognitive impairment, an uncontrolled psychiatric condition, geographic inaccessibility); contraindication to PVP surgery; or contraindication to general anaesthesia.

### Randomization

The surgeon will perform patient randomization at the time of the inclusion visit. The numbers of the two strategy groups will be balanced with a ratio of 1:1. Randomization will be stratified by the investigation centre and by the indwelling catheter at the time of surgery, which is known to be an independent factor of perioperative morbidity [15]. The randomization will be performed by a central randomization system, and allocation of the strategy will be performed by a minimization method to ensure well-balanced groups [16].

### Study intervention

#### Surgical procedure

The elective surgical procedure will be performed after approval of the surgeon and anaesthesiologist, and the surgery will be performed according to the usual indications of the PVP technique. The surgical procedure will be performed under general anaesthesia, owing to the OAC treatment. PVP will be carried out according to the technique described by Malek et al. [17] using a GreenLight XPS 180-W device (Boston Scientific Corporation) and a MoXy fibre inserted through the working channel of a continuous double-flow 23-Ch or 26-Ch cystoscope with 0.9% saline irrigation. Urine sterility will be analysed before the surgery. Asymptomatic bacteriuria (with or without an indwelling catheter) will be treated with an appropriate antibiotic for at least 48 h before the surgery. If a case of urethral meatus stenosis makes the introduction of the cystoscope difficult, atraumatic dilatation will be preferred rather than performing an urethrotomy to avoid bleeding in the patients under ongoing OAC treatment.

#### Management of anticoagulation treatment during the perioperative course

**Fig. 1:**
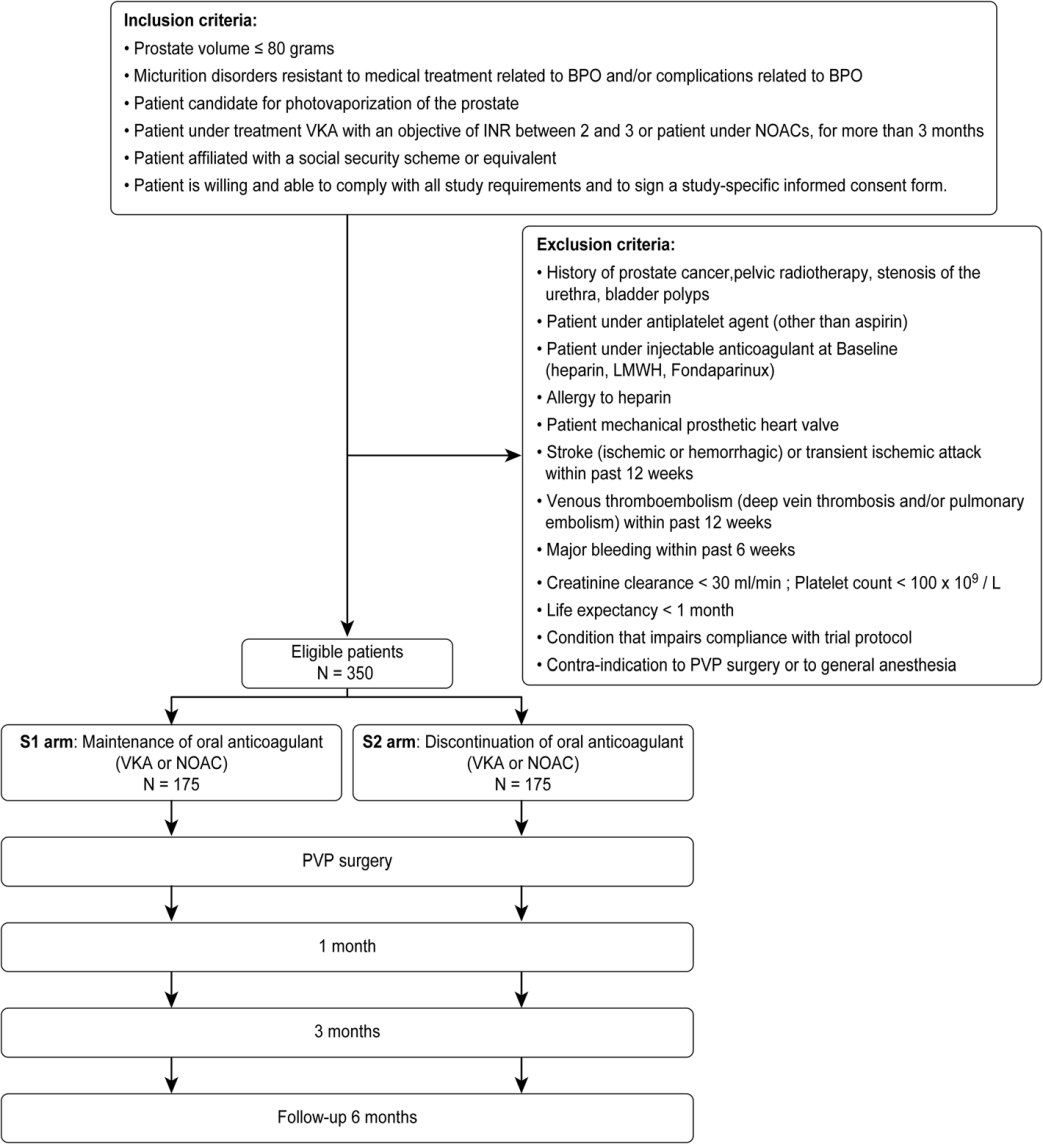
Flow diagram of the study design. Patients will be randomly allocated in a 1:1 ratio to the OAC group (S1) or the control group (S2)

This study focuses on perioperative OAC management for BPO surgery with PVP (Fig. 1). Only OAC therapies, including VKAs and DOACs, are considered in this trial. Patients taking antiplatelet agents (other than aspirin) will be excluded.

**Fig. 2:**
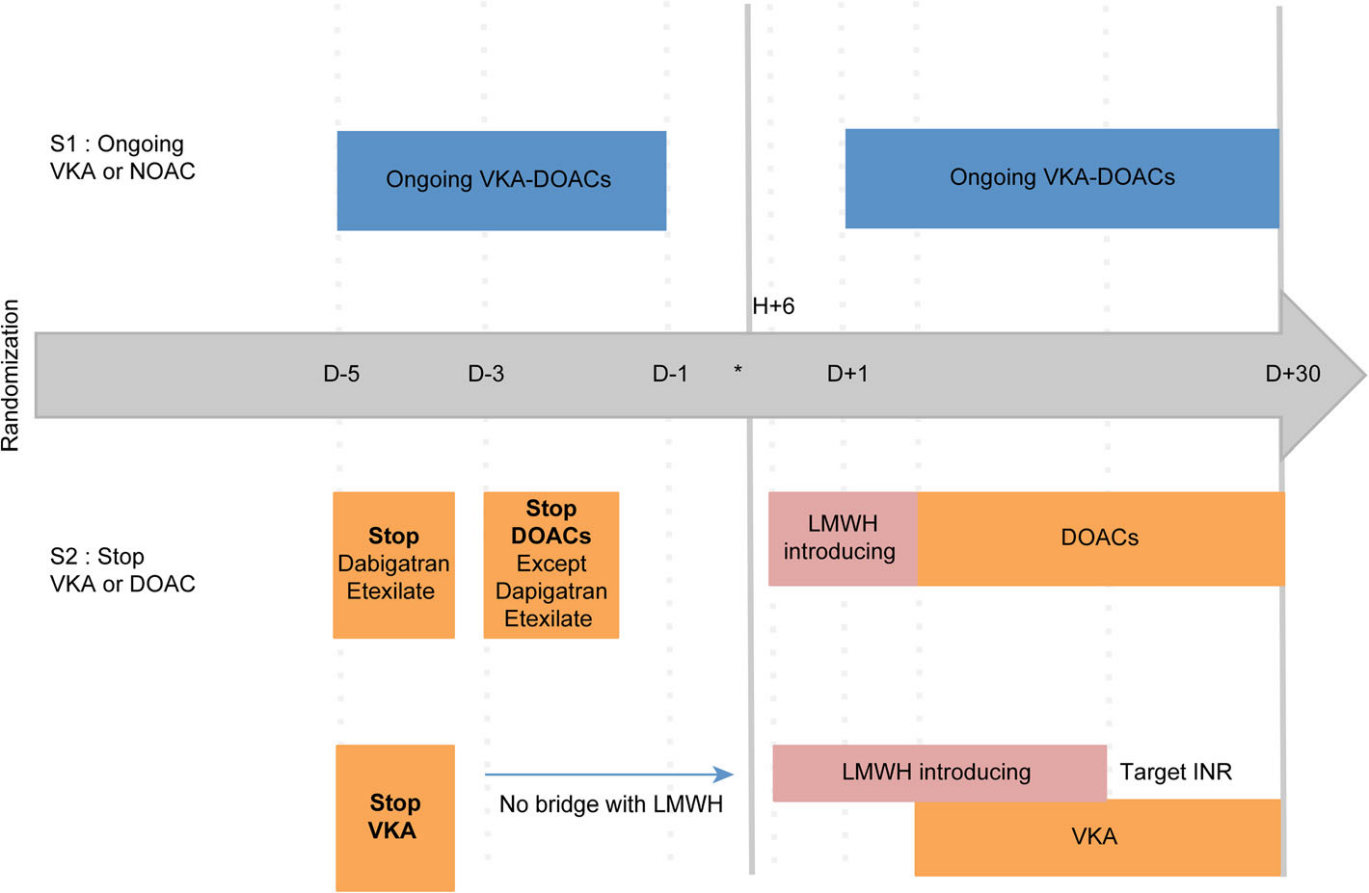
Study protocol

Both strategies are presented in Fig. 2. The interventional group (S1) includes patients taking an OAC until the day of the surgery. OAC treatment will be resumed the day after surgery. The control group (S2) includes patients who will stop OAC treatment before surgery according to the French anaesthesiology guidelines, which state that if a patient is taking a VKA or dabigatran, treatment will be stopped 5 days before the procedure. If the patient is treated with a DOAC other than dabigatran, treatment will be stopped 3 days before PVP surgery. The postoperative prescription of a prophylactic or a curative dose of LMWH is left to the discretion of the anaesthesiologist. OAC treatment will be resumed when haemostasis is achieved, which will be judged as appropriate and safe enough by the surgeon. However, we recommend resuming OAC treatment as early as possible in the postoperative course.

### Data collection and follow-up

The patients’ baseline demographics and medical history will be collected. All patient data will be collected on an electronic case report form (eCRF). The following data will be collected as scheduled (see Table 1): consent, complete biological assessments, urinary culture analysis, prostate-specific antigen (PSA) level, prostatic ultrasound measurement, uroflowmetry (Qmax), IPSS, Urinary Symptom Profile (USP) score, CHA_2_DS_2_-VASc score, HAS-BLED score, ATRIA bleeding risk score, treatments including OAC treatment, intraoperative data and immediate postoperative AEs.

**Table 1:** Standard protocol items

	V1 Baseline M-1	Hospitalization			V2 1 month follow-up +/− 7 days	V3 3 months follow-up +/− 7 days	V4 6 months follow-up +/− 7 days
		D-1	Discharge	D0			
Patient information	✓						
Informed consent	✓						
Verification of eligibility criteria	✓						
Randomization	✓						
Clinical examination	✓					✓	✓
Complete biological check-up		✓			✓		
CBEU	✓				✓	✓	✓
PSA	✓					✓	✓
Ultrasound: measurement of prostatic volume and post-voiding residual	✓					✓	
Uroflowmetry: measurement of Qmax	✓					✓	✓
IPSS and USP questionnaires	✓				✓	✓	✓
Collection of patient data	✓						
Collection of operative data				✓			
Scores: CHA_2_DS_2_-VASc, HAS-BLED, ATRIA bleeding risk		✓			✓		
Collection of treatments	✓			✓	✓	✓	✓
Collection of adverse events			✓	✓	✓	✓	✓

Patients will be followed up to 6 months after PVP surgery, with three visits scheduled at 1 month, 3 months and 6 months after the surgery. The urologists will perform the follow-up and will be responsible for collecting all of the data needed for the study, including data on any AEs that will be classified according to the Clavien-Dindo classification.

Monitoring visits will be scheduled in the centres to verify patient consent, protocol compliance and data quality. These visits will be adapted to the rhythm of the included centres.

### Outcome measures

The primary outcome is the number of patients with at least one complication classified greater than or equal to grade 2 according to the Clavien-Dindo classification at the 30 days follow-up. An independent adjudication committee composed of two surgical urologists will meet every 6 months to review the AEs and to validate the judgement criteria. This committee will be blinded to the randomization.

Secondary outcomes are the number of patients with at least one complication classified as greater than or equal to grade 2 according to the Clavien-Dindo classification at 6 months and the number of patients with at least one haemorrhagic or thrombotic complication at 1 month and 6 months. Moreover, the duration of catheterization, the length of hospital stay, the prostatic residual volume, PSA level, IPSS score, IPSS question 8 and USP, uroflowmetry (Qmax) and post-voiding residual will be collect during the follow-up visits.

Bleeding complications are defined as follows: a significant and persistent haematuria in a patient with or without a urethral catheter (Clavien 2) leading to a decrease in haemoglobin of 2 g/dl or more; a gross haematuria requiring clot removal through the bladder catheter under local anaesthesia (Clavien 3a) or with a cystoscope under general anaesthesia (Clavien 3b); and bleeding appearing in the immediate postoperative course, with the requirement of a red blood cell transfusion (Clavien 2).

As the Clavien-Dindo classification is not accurate enough to report postoperative thrombotic events, we choose the same definition of thrombotic complications described in the BRIDGE study [18]. The first is a stroke, where either of the following criteria must be satisfied: any new, focal neurologic deficit that persists for > 24 h or any new, focal neurologic deficit of any duration and with evidence of acute infarction on computed tomography (CT) or magnetic resonance imaging (MRI) of the brain. The second is a TIA, where the following criteria must be satisfied: any brief neurologic deficit caused by focal brain or retinal ischaemia, with clinical symptoms lasting, typically, for < 1 h and not for > 24 h; no evidence of acute infarction on CT or MRI of the brain; and no signs of acute myocardial infarction on CT scan or MRI. The third thrombotic complication is a systemic embolism, where all three of the following criteria must be satisfied: a symptomatic embolic episode associated with an abrupt arterial insufficiency to the upper extremity, lower extremity or abdominal visceral organ; the embolism is verified by intraoperative or radiologic evidence (e.g. CT angiography) of an arterial occlusion; and the embolism occurs in the absence of other likely mechanisms (e.g. atherosclerosis).

### Sample size estimation

The main objective of our study is to assess the non-inferiority of the risk of complications while maintaining OAC treatment in patients with BPO who are undergoing surgery via GreenLight PVP. The risks of complication are assessed using the following binary criteria:

- Complication = 1 if at least one complication is classified as ≥2 according to the Clavien-Dindo classification
- Complication = 0 if the only complications that occur are classified as < 2 according to the Clavien-Dindo classification.

Our literature analysis failed to find published references on the risks of complications for any of the two management strategies assessed in the current study. However, the analysis of a single-centre PVP database of the Clinique Pasteur (Toulouse, France) makes it possible to estimate the proportion of patients presenting with “complication = 1”. This estimated risk is 13.2% (95% confidence interval [0.091, 0.183]). The assumption of our study is that this proportion of 13.2%, which was obtained on the basis of patients of the Clinic Pasteur, will be obtained for each of the two strategies under study. The margin of non-inferiority will be set at 10%, which is clinically reasonable. The value of the non-inferiority margin was defined after a non-informal survey of clinical experts who have experimented with both intervention strategies.

Using two-tailed 95% confidence intervals, non-inferiority would be identified with 80% power up to a non-inferiority margin of 10% if approximately 350 participants are randomized to the study. Thirty-nine patients will be added to account for potential withdrawals during the trial.

### Statistical methods

All statistical analyses will be thoroughly performed by independent statisticians who are not involved in patient treatment or outcome assessment. The statisticians will be blinded to the allocation code. The statisticians will perform statistical analyses according to predetermined data handling and statistical methods, and there will be no arbitrary interference. All analyses will be performed on per-protocol (PP) and intention-to-treat (ITT) populations in accordance with the recommendations on non-inferiority trials [19].

Baseline characteristics by group will be compared using descriptive analyses.

For the primary endpoint, the judgement criterion analysis will be performed using the non-inferiority confidence intervals approach. These analyses will be carried out on the per-protocol population (patients with no major deviation from the protocol that may affect the assessment of the primary endpoint) and the ITT model (ITT-m) population (ITT and underwent surgery).

Assessments of the quantitative criteria will be carried out by means of *t* tests if the distribution permits or by non-parametric methods (Wilcoxon-Mann-Whitney test). Assessments of the qualitative criteria will be made by means of proportional comparisons via chi-square tests. Subgroup assessments will be considered based on stratification factors of randomization (i.e. the centres and the presence of indwelling catheter at baseline).

To evaluate non-inferiority, the hypothesis testing strategy will follow the two one-sided tests (TOST) procedure developed by Schuirmann [20]. To assess superiority, the hypothesis testing strategy will be two-tailed. A *P* value of 0.05 will be considered statistically significant. All data analyses will be performed using SAS® (version 9.4 or later) or R.

### Interim analysis

No interim analysis was planned in the study protocol.

### Data and safety monitoring committee

This study focuses on an assessment of two commonly used strategies for which the risk level is very limited, and an independent monitoring committee is not required.

### Auditing

An audit may be carried out at any time by persons appointed by the sponsor who are independent of the team responsible for the research. The purpose of an audit is to ensure the quality of the research, the validity of the results, compliance with the law and the application of regulations.

## Discussion

OAC treatments, and particularly DOAC therapies, expose patients to a high risk of bleeding. PVP has emerged in the last decade to treat frail patients and is now considered as an effective option for patients under ongoing OAC therapy who require surgical treatment for symptomatic BPO. However, only a few retrospective studies with little evidence have reported on PVP in patients under ongoing OAC therapy. The SOAP trial will be the first multicentre RCT to evaluate the safety and efficacy of PVP in patients under ongoing OAC therapy.

The use of OACs has been steadily increasing since 2008, mainly due to the emergence of DOACs. Thus, more than 150 million OAC doses are consumed daily worldwide; nearly 1.4 million people are taking an OAC, and 40% of them are 80 years old or older. Among these patients, 15–20% will undergo an invasive procedure or surgery that interrupts their chronic OAC treatment, putting them at increased risk for thromboembolism, haemorrhage and death [21]. Perioperative anticoagulation management is a common clinical dilemma, often leading to significant AEs. The clinical relevance of this dilemma and the lack of definitive evidence to guide medical decisions have led to the realization of this pertinent RCT concerning the surgical management of BPO.

To assess the routine practice of BPO surgeries in patients under ongoing OAC therapy, an Internet survey was sent by Becker et al. to all active members of the Endourological Society [22]. Among all respondents, 18% indicated that they perform transurethral surgeries in patients under ongoing OAC therapy, whereas 60% of this group indicated that they temporarily stop the OAC therapy during the intervention. Furthermore, 16% of the respondents perform more than 30 transurethral interventions per year for BPO patients on ongoing OAC therapy. Most procedures were performed under aspirin (58.2%). Treatments with adenosine diphosphate (ADP) receptor inhibitors (22.1%), VKAs (18.9%), factor Xa inhibitors (15.6%) or a combination of two OACs (16.4%) were continued less often than those performed under aspirin. In this worldwide questionnaire, the GreenLight laser (39%) was the most frequently used technique for patients under ongoing OAC treatment, followed by bipolar TURP (35%) as well as other sources of laser (holmium laser [12%], thulium laser [12%] and diode laser [2%]). Despite the small amount of evidence, Becker et al. have shown that transurethral surgeries for BPO patients under OAC therapy were routinely performed, especially with the GreenLight laser.

The only RCT investigating the 180-W XPS GreenLight laser for PVP showed a non-inferiority of PVP to TURP for functional outcomes and complication rates [6]. However, patients were discontinued from OAC therapy preoperatively for 3–5 days and were excluded if they were unable to do so. In other studies addressing the safety and efficacy of the 180-W laser, patients on OACs were included in the cohort, but no specific comparisons were made [23, 24]. The largest retrospective series was carried out on 59 patients under OAC therapy who underwent PVP, reported by Knapp et al. [11]. However, according to the subgroup analysis, only 32 patients were treated with an OAC (23 with VKAs and 9 with DOACs). Among the patients included in the OAC group, 39% received platelet inhibitors including clopidogrel (20), a combination of dipyridamole and aspirin (1) or a combination of clopidogrel and warfarin (2). Thus, it is unclear if the difference reported between the OAC group and the control group regarding the number of high-grade AEs was related to platelet inhibitors or OACs. Moreover, it seems that high-grade AEs were not bleeding complications but mainly infectious complications likely due to the low rate of frail patients included in this study.

In this trial, only VKAs and DOACs are considered. Patients treated with antiplatelet agents (other than aspirin) are excluded. It is paramount to distinguish OACs from platelet inhibitors when assessing PVP postoperative complications. Indeed, the quality of haemostasis must be explained by the mechanism of coagulation provided by the PVP procedure. The energy from a 532-nm-wavelength potassium titanyl phosphate (KTP) laser is absorbed by haemoglobin, and the heat is concentrated into a small volume, lysing the tissue by rapidly vaporizing cellular water. Only a 2-mm rim of coagulated tissue is vaporized [25]. The efficacy of PVP haemostasis is probably not comparable between antiplatelet therapies acting via primary haemostasis and OACs acting via coagulation mechanisms. It is therefore desirable to distinguish the management of patients under OAC treatment or under platelet inhibitors treatment during the perioperative course of PVP surgery for BPO.

### Trial status and timeline

As of 30 September 2018, 30 patients were enrolled, and recruitment is ongoing. Approximately 11 institutions are preparing to begin patient enrolment. A total of 386 patients will be recruited for the trial within 2 years.

The recruitment of patients started in January 2018 and will finish in January 2020.

## Abbreviations

BPO: Benign prostatic obstruction
DOAC: Direct oral anticoagulant
FNAH: French National Authority for Health
INR: International normalized ratio
ITT-m: Intention-to-treat model
LMWH: Low-molecular-weight heparin
OAC: Oral anticoagulant
PVP: Photoselective vaporization of the prostate
TIA: Transient ischaemic attack
TURP: Transurethral resection of the prostate
VKA: Vitamin K antagonist
